# Distinct Compartmentalization of Microbial Community and Potential Metabolic Function in the Fruiting Body of *Tricholoma matsutake*

**DOI:** 10.3390/jof7080586

**Published:** 2021-07-22

**Authors:** Dong Liu, Jesús Perez-Moreno, Peng Zhang, Ran Wang, Caspar C. C. Chater, Fuqiang Yu

**Affiliations:** 1 The Germplasm Bank of Wild Species, Yunnan Key Laboratory for Fungal Diversity and Green Development, Kunming Institute of Botany, Chinese Academy of Sciences, Kunming 650201, China; liudongc@mail.kib.ac.cn (D.L.); zhangpeng@mail.kib.ac.cn (P.Z.); wangran@mail.kib.ac.cn (R.W.); 2 Colegio de Postgraduados, Campus Montecillo, Microbiología, Edafología, Texcoco 56230, Mexico; jepemo@yahoo.com.mx; 3 Department of Crop and Forest Science, University of Lleida, Av. Alcalde Rovira Roure, 191, 25198 Lleida, Spain; 4 Department of Natural Capital and Plant Health, Royal Botanic Gardens, Kew, Richmond, Surrey TW9 3AE, UK; caspar.chater@gmail.com

**Keywords:** *Tricholoma matsutake*, ectomycorrhizal fungi, microbial partitioning, metabolic profiling, Basidiomycota, fruiting body

## Abstract

The uniquely compartmentalized fruiting body structure of the ectomycorrhizal fungus (EMF) *Tricholoma matsutake*, is a hotspot of microbial habitation and interaction. However, microbial diversity within this microniche structure of the EMF is rarely investigated. Furthermore, there is limited information concerning microbiomes associated with sporomes belonging to the ubiquitous fungal phylum Basidiomycota, particularly with respect to fungus-EMF interactions. In this study, we conducted high throughput sequencing, using ITS (fungal) and 16S rRNA (bacterial) marker genes to characterize and compare fruiting body microbiomes in the outer (pileipellis and stipitipellis) and inner layers (pileum context, stipe context, and lamellae) of the fruiting body of *T. matsutake*. Our results show the number of unique bacterial operational taxonomic units (OTUs) among the different compartments ranged from 410 to 499 and was more than double that of the shared/common OTUs (235). Micrococcales, Bacillales, *Caulobacter*, and *Sphingomonas* were the primary significant bacterial taxa within the different compartments of the dissected *T. matsutake* fruiting body. Non-parametric multivariate analysis of variance showed significant compartmental differences for both the bacterial and the fungal community structure within the *T. matsutake* fruiting body. The metabolic profiling revealed putative metabolisms (of amino acids, carbohydrates, and nucleotides) and the biosynthesis of secondary metabolites to be highly enriched in outer layers; in the inner parts, the metabolisms of energy, cofactors, vitamins, and lipids were significantly higher. This study demonstrates for the first time the distinct compartmentalization of microbial communities and potential metabolic function profiles in the fruiting body of an economically important EMF *T. matsutake*.

## 1. Introduction

The fungal phylum Basidiomycota is one of the most diverse fungal groups, with a presence over a wide environmental range [1]. Nearly 31,000 species across different genera belong to the phylum and account for more than a quarter of all known species of Eumycota [2]. Basidiomycota play a crucial role in terrestrial ecosystems, with members belonging to important trophic modes such as the saprotrophs, pathogens, or ectomycorrhizal fungi (EMF) [3,4]. Dominant in forest ecosystems, EMF regulate plant health, nutrient exchange, and forest sustainability [5,6]. Many studies in recent years have demonstrated important microbial interactions during the process of EMF development and establishment. Some of these include facilitating mycorrhizal formation, promotion of mycelial growth, and increasing EMF nutrient foraging ability, as well as basidiomata formation [7].

Completing their life cycle, the EMF release spores from the fruiting bodies, which also nutritionally support interacting microbial groups such as bacteria [8]. These endofungal bacteria occur widely within EMF fruiting bodies and are selected from the environment based on their symbiotic functions or habitat requirements [9]. Such endofungal bacteria can promote fruiting body formation and/or compartmentalization in the EMF, as can be seen with *Bradyrhizobium*, *Roseiarcus*, and *Pseudolabrys* during primodium formation and fruiting body development of *Phlebopus portentosus* (Boletales, Basidiomycota) [10]. On the other hand, some endofungal bacteria (e.g., *Ewingella*, *Pseudomonas*, and *Stenotrophomonas*) may acquire their nutrition by acting as “decomposers” of fungal fruiting bodies [11] and mycelia [12,13]. Unlike bacterial-fungal interactions, very little attention has been given to yeasts or filamentous fungi associated with basidiomata. To date, there is only one study related to the fungal microbiome distribution in the microniches of basidiomata-forming fungi [3]. Using functional gene analysis, Liu et al. (2021) found that *Monographella*, *Scytalidium*, and *Vanrija* were correlated with functional gene structure in the context; *Aspergillus* and *Lecanicillium* were associated with the hymenophore; and *Trichosporon* were significantly (*p* < 0.01) associated with the pileipellis of *Thelephora ganbajun* (Thelephorales, Basidiomycota) [3]. 

So far, the microbiome associated with the microniches of the Basidiomycota order Agaricales has not been studied. Agaricales, also known as gilled mushrooms (for their distinctive gills) or euagarics, contains some of the most familiar types of mushrooms. The order contains 33 extant families, 413 genera, and over 13,000 described species [1]. To begin to address this vast knowledge gap, we ask:

(i) If there are differences in specific bacterial and fungal taxa associated with the compartments of Agaricales;
(ii) What are the dominant potential metabolic functional traits in different compartments of sporophores of Agaricales?

To seek answers to such intriguing questions, we selected *Tricholoma matsutake* (Tricholomataceae, Agaricales, Basidiomycota) as a model fungus owing to the facts that:

(a) It is an iconic Agaricales mushroom species which belongs to Basidiomycota, with distinct fruiting body structures [14];
(b) It is ecologically important in maintaining forest health and functioning, through formation of symbiotic ectomycorrhizal relationships with forest trees species worldwide such as *Picea glehnii* in Japan, *Pinus densiflora* in Korea, and *P. sylvestris* in Europe, as well as *Castanopsis*, *Quercus*, and *Lithocarpus* in China [15,16].

**Figure 1:**
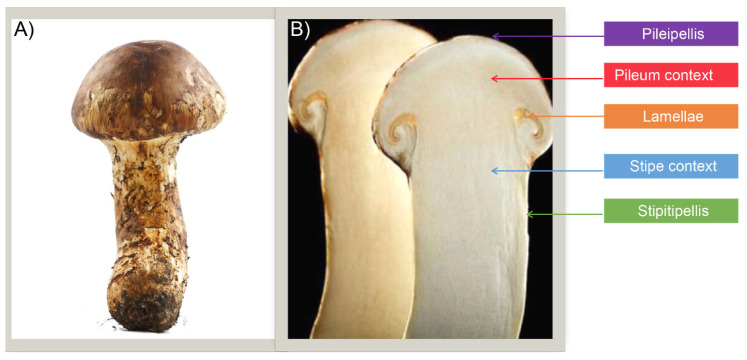
Basidiomata of *Tricholoma matsutake*: lateral (**A**) and cross section diagram (**B**), showing the positions of the five compartments. For further details of the compartment sampling please refer to Section 2.2.

Five compartments were selected according to *T. matsutake* fruiting body structure, from their outer (pileipellis and stipitipellis) and inner (pileus context, stipe context, and lamellae) layers (as shown in Figure 1). Therefore, the microbiome of *T. matsutake* was analyzed through high-throughput amplicon sequencing to assess whether compartments influence the structure and diversity of bacterial and fungal communities within *T. matsutake* fruiting bodies. To the best of our knowledge, this is the first study to explore the microbial diversity and specificity of microbiomes in different compartments of an Agaricales species. 

We hypothesized that the bacteria and fungi would be selective in inhabiting the different *T. matsutake* fruiting body compartments, which would be reflected by a diverse microbiome and community structure in different compartments of *T. matsutake* (H1). As a consequence of this differential microbiome composition, we further hypothesized that potential microbial metabolic functions would vary significantly between different compartments of the *T. matsutake* fruiting body (H2). To predict the functional capability within a metagenome, we used a database of reference genomes, marker genes, and a computational simulation method. This bioinformatics approach, widely used in recent microbial ecology studies, is referred to as the Phylogenetic Investigation of Communities by Reconstruction of Unobserved States (PICRUSt) [17,18,19].

## 2. Materials and Methods 

### 2.1. Site Description and Sampling Method

*Tricholoma matsutake* fruiting bodies were collected from a *Pinus yunnanensis* forest near Lu Quan County, Yunnan province, Southwest China (25°25′48′′ N, 102°13′12′′ E, 2176 m above sea level). Chemical characterization of the sampling site showed that the forest soil was acidic (pH 4–5), with high organic matter content (>40 g kg^−1^), and an exchangeable Ca^2+^ concentration of >700 mg kg^−1^. Daily variation of the soil and air temperatures ranged between 14.1–14.3 °C and 19.1–24.4 °C, respectively. Daily air moisture was 50–63%, and soil water content changed between 18% and 29% at the sampling sites. The fruiting bodies were collected during their fruiting season on 25 October 2018. In order to obtain ecologically independent samples, three adjacent hills were selected as sampling areas. At each hill, different sampling points at different slope aspects, but similar altitude, were separated by more than 200 m for each *Tricholoma matsutake* fruiting body to minimize the heterogeneity of subplot effects. More than fifteen fruiting bodies were obtained and from them six immature ones were chosen as representative samples (in order to reduce probability of microbial contamination from environmental factors such as wind and rain) and used for subsequent compartment separation (Figure 1). 

In total, there were three ecological replicates per compartment for a total of 5 (compartment) × 3 (ecological independent hill) = 15 samples.

### 2.2. Mushroom Fruiting Body Compartment Separation

Field-fresh basidiomata were collected using sterile latex gloves in order to avoid contamination. The selected basidiomata were individually wrapped in aluminum foil and stored in separate sterile bags and transported in iceboxes to the laboratory within 12 h. Before tissue separation, the fruiting bodies were surface-cleaned with sterilized milli-Q water and dried with sterilized absorbent paper, as described by Liu et al. (2021). In the lab, the stipe base was cut with a sterile scalpel and only the upper and middle areas of the stipe were analyzed in order to avoid contamination with soil microbes adhered to the stipe base. After that, all the fruiting bodies were separated under axenic conditions, using sterile scalpels, into five compartments according to *T. matsutake* distinct fruiting body structures: L = lamellae; P = pileipellis; PC = pileum context; S = stipitipellis; SC = stipe context. For didactic purposes, these compartments that made up the microniches analyzed are shown in Figure 1. All these sub-samples were then immediately placed in sterile self-sealing bags (60 mm × 85 mm) at −20 °C prior to microbial analysis.

### 2.3. DNA Extraction and PCR Amplification

Genomic DNA was extracted from 0.4 g of each *T. matsutake* compartment, using the DNeasy Plant Mini Kit (Qiagen, Hilden, Germany) following the manufacturer’s instructions. The V4 hypervariable region of the bacterial *16S* rRNA gene was amplified with the forward primer 502F (5′-AYTGGGYDTAAAGNG-3′) and the reverse primer 802R (5′-TACNVGGGTATCTAATCC-3′). *Internal transcribed spacer 2* (*ITS 2*) was used for fungal community analyses, using the primers ITS5-1737F and ITS2-2043R [20,21]. PCR thermal cycling condition were as described in Xiong et al. (2016) [22]. Amplicons were extracted from 1% agarose gels and purified using the EZNA Gel Extraction Kit (OMEGA bio-tek, Doraville, GA, USA), and then quantified on a Microplate reader (BioTek, Winooski, VT, USA, FLx800) using the Quant-iT PicoGreen dsDNA Assay Kit, Invitrogen (P7589, Carlsbad, CA, USA). In addition, ITS analysis was conducted to molecularly confirm the identification of the studied *T. matsutake* samples. Briefly, the *internal transcribed spacer 1* (*ITS 1*) was used for fungal identification, with primers ITS5F (5′-GGAAGTAAAAGTCGTAACAAGG-3′) and ITS1R (5′-GCTGCGTTCTTCATCGATGC-3′) (Chang et al., 2001) [23]. PCR products were purified using a purification kit (QIAquick PCR purification kit; Qiagen, Hilden, Germany), and sequencing was performed on an ABI 3730XL automatic sequencer (Sunny Biotech Company, Shanghai, China). The obtained nucleotide sequences were deposited in the GenBank database with accession numbers MT126627-126630. 

### 2.4. Illumina Miseq Sequencing and Data Processing

Sequencing libraries were generated using NEBNext^®^ Ultra™ DNA Library Prep Kit for Illumina^®^ (New England Biolabs, Ipswich, MA, UK) with index codes. The library was sequenced on an Illumina MiSeq platform generating 2 × 300 bp paired-end reads (Personalbio Co., Ltd., Shanghai, China). Sequences were processed following these steps: (i) raw read quality control and paired-end clean read assembly; and (ii) raw tag quality control. During high-throughput sequencing, erroneous or questionable sequences can occur; QIIME software (Quantitative Insights into Microbial Ecology, V1.8.0, http://qiime.org/, accessed on 3 January 2019) [24] was used to identify these. In addition to a requirement for >160 bp sequence lengths and the exclusion of fuzzy base N, we also excluded: (1) sequences with a mismatched base number >1 of the 5′ end primer; and (2) sequences with >8 identical consecutive bases. Then, obtained sequences were processed with the USEARCH software (http://www.drive5.com/usearch/, accessed on 5 February 2019) to check and remove chimeric sequences. Finally, the cleaned high-quality sequences were classified into operational taxonomic units (OTUs) at a 97% similarity cutoff. The most frequently occurring sequence was kept as a representative sequence for each OTU and selected for annotation. The SILVA (for 16S, https://www.arb-silva.de/, accessed on 9 February 2019) and Unite (for ITS, http://unite.ut.ee/index.php, accessed on 9 February 2019) databases were used to annotate taxonomic information. High quality sequences in the lengths of 345–409 bp (16S) and 200–437 bp (ITS), were reserved for the taxonomic level analysis. In the fungal ITS sequence analyses, to exclude host fungus interference, *T. matsutake* ITS sequences (559 OTUs, accounting for 63% of the total fungal OTUs), were filtered out from the data for downstream analyses. Finally, the resulting OTUs were assigned to their functional groups based on PICRUSt [18] and the FUNGuild tool [25] for bacteria and fungi, respectively.

Raw sequence data from this study were deposited in the NCBI Sequence Read Archive under the accession number PRJNA609601.

### 2.5. Statistical Analysis 

Microbial alpha-diversity was estimated by richness (Chao 1 and ACE) and diversity (Shannon and Simpson) indexes. One-way analysis of variance followed by Tukey HSD (at *p* < 0.05) was used to compare significance. The shared or unique microbial OTU numbers (among different fungal compartments) were calculated based on the OTU abundance matrix using (nVennR) package in R software. Dissimilarity of microbial community matrix (beta-diversity) was evaluated using the pairwise UniFrac distance [26] and visualized via the unweighted non-metric multi-dimensional scaling plots (NMDS) in Origin 2018 software. Significant differences of the UniFrac distance matrixes among the five *T. matsutake* compartments were tested using nonparametric multivariate analysis of variance (ADONIS) and analysis of similarities (ANOSIM) within the vegan package in R (O’Connor, 1988). Linear discriminant analysis (LDA) effect size (LEfSe) was used to investigate important biomarkers of microbial communities (LDA score > 2.0 and *p* < 0.05) that are specifically enriched in compartments of *T. matsutake*. The cladogram was visualized with the LEfSe algorithm using the Huttenhower Galaxy web application [27]. A computational approach—Phylogenetic Investigation of Communities by Reconstruction of Unobserved States (PICRUSt) was used to predict a community’s metabolism functions [18].

## 3. Results

### 3.1. Microbial Diversity Differences in Various Compartments

A total of 600,817 (*16S* sequences) and 467,430 (*ITS* sequences) for bacterial and fungal communities were obtained from the 5 *Tricholoma matsutake* fruiting body compartments. Cleaned reads of bacterial OTUs ranged from 31,695 to 45,509 and were rarefied to 31,695 sequences per sample. Cleaned reads of fungal OTUs ranged from 27,248 to 37,235 and were rarefied to 27,248 sequences per sample. Detailed sequence numbers in individual samples can be found in Table S1.

**Figure 2:**
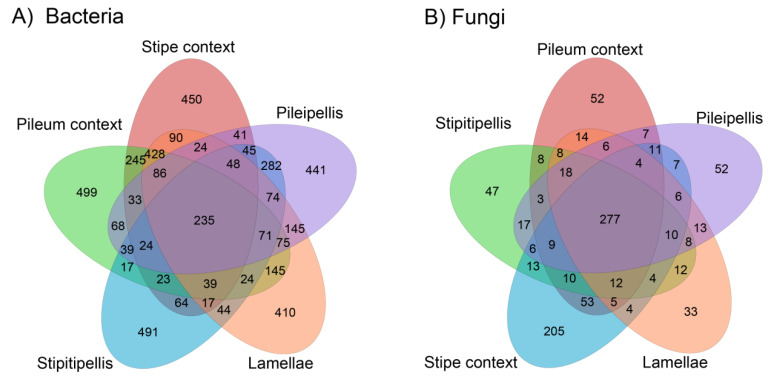
Venn diagrams of shared or unique (**A**) bacterial and (**B**) fungal OTUs among the different compartments of *Tricholoma matsutake* fruiting bodies. The microbial taxa of these OTU data can be found in Table S2 (Bacterial OTUs) and Table S3 (Fungal OTUs).

Bacterial diversity indices (Simpson and Shannon) exhibited no significant differences among *T. matsutake* compartments, but bacterial richness indices (as revealed by Chao 1 and ACE; Table 1) were significantly highest in the pileum context, which was in accordance with the highest number of unique bacterial OTUs found there (499; Figure 2A). Among different *T. matsutake* tissues, the bacterial richness was higher in the inner layer (pileum context > stipe context) than in the outer layer (pileipellis > stipitipellis) (Table 1).

Differences in *T. matsutake* fungal communities showed the opposite trend, with similar richness, but significant differences in diversity indices: significantly higher in the stipe context than in other compartments (Table 1). This difference was in line with the highest number of unique fungal OTUs found in the stipe context (205; Figure 2B).

The different compartments of the *T. matsutake* fruiting body shared a similar unique number of either bacterial or fungal OTUs, except for the stipe context (Figure 2). Noticeably, the unique fungal OTUs were around four times higher in the stipe context than in other *T. matsutake* tissues (Figure 2B). The number of shared *T. matsutake* bacterial and fungal OTUs was comparable (235 vs. 277), but the total unique bacterial OTUs (2291) was approximately six times higher than that of the fungal OTUs (389) (Figure 2).

### 3.2. Differences in Microbial Relative Abundance and Community Structure

**Figure 3:**
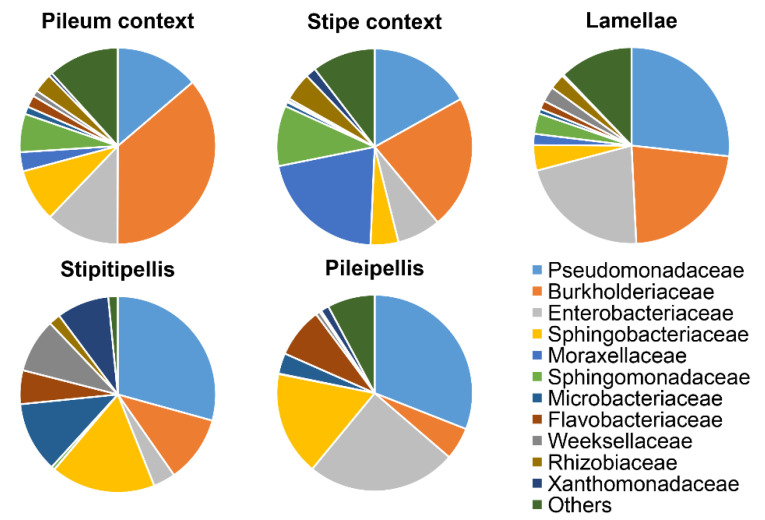
Relative abundance of the top 11 bacterial families found in different compartments associated with *Tricholoma matsutake* fruiting bodies.

For bacteria, all the *T. matsutake* compartments were dominated by Proteobacteria (76% of all OTUs) and Bacteroidetes (17%), followed by Actinobacteria (4.6%), Firmicutes (1.2%), Deinococcus-Thermus (0.5%), and Acidobacteria (0.4%) (Figure S1). The relative abundance of Proteobacteria was higher in the inner layer (averaged 84%) of the *T. matsutake* fruiting bodies (pileum context, lamellae and stipe context) than in the outer layer (averaged 62% in pileipellis and stipitipellis). Contrastingly, Bacteroidetes preferred to colonize the outer layer of *T. matsutake* (~30% relative abundance) rather than the inner layer (~8%). The family Burkholderiaceae was over three times higher in the contexts of pileum and stipe (inner layer; ~27% relative abundance) than in the other compartments (~8%) (Figure 3). The relative of abundance of Pseudomonadaceae (~30%) and Sphingobacteriaceae (17%) was almost doubled in the outer layer (stipitipellis and pileipellis) than in the contexts (~15% of Pseudomonadaceae; ~7% of Sphingobacteriaceae). Of particular interest, Moraxellaceae has limited abundance in all compartments (ranged from 0.01%~3%; Figure 3), except for in the stipe context (21%). 

The *T. matsutake* endofungal phyla were dominated by Basidiomycota (91.2%), followed by Ascomycota (6.8%), Zygomycota (0.02%), Rozellomycota (0.01%), Chytridiomycota (0.01%), and Glomeromycota (0.002%) (Figure S2). Within the phylum Basidiomycota, the family Thelephoraceae preferred the contexts (~12%, relative abundance) to other compartments (Figure S3), whereas fungal families Dothioraceae, Trichocomaceae, and Hypocreaceae (belonging to the Ascomycota) had higher relative abundance in the outer layer of *T. matsutake* (~5%) than in the contexts (<0.3%) (Figure S3).

**Figure 4:**
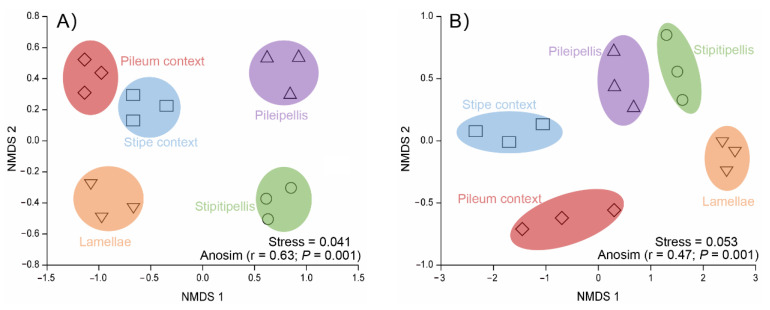
Bacterial (**A**) and fungal (**B**) community composition as indicated by unweighted non-metric multi-dimensional scaling plots (NMDS) of pairwise UniFrac community distance across the 15 *Tricholoma matsutake* compartment samples. The distance-based results of permutation dissimilarity tests are displayed on the bottom right corner of each graph, including non-parametric multivariate analysis of variance (ADONIS) using distance matrices and the analysis of similarity (ANOSIM).

In order to compare the beta-diversity of *T. matsutake*-inhabiting microbial communities, non-metric multi-dimensional scaling analyses (NMDS) were performed for bacterial (Figure 4A) and fungal OTUs (Figure 4B). Both bacterial and fungal community structure of *T. matsutake* showed significant differences in various compartments (ANOSIM test, *p* < 0.01). The *T. matsutake* microbial community structure differed more for bacteria (stress = 0.041) than for fungi (stress = 0.053), which was in line with the higher number of unique bacterial OTUs in different compartments (Figure 2). Therefore, we further investigated the bacterial biomarkers in various *T. matsutake* compartments.

### 3.3. Primary Taxa Difference Analysis 

**Figure 5:**
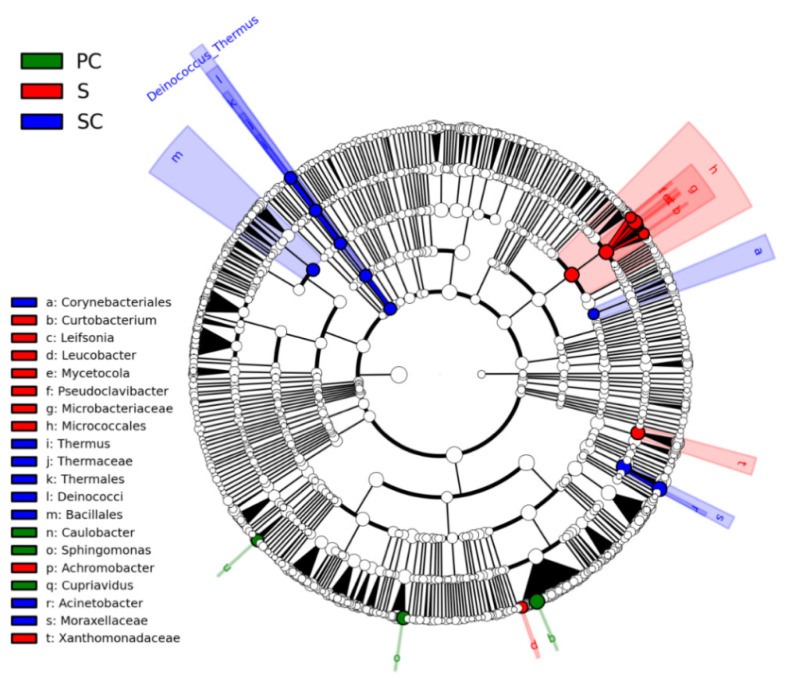
Taxonomic cladogram generated using Lefse analysis. The genus, family, order, class and phylum are listed in order from the outside (big circle) to the inside (small circle) of the cladogram. Green, red, and blue indicate taxa enriched in the pileum context (PC), stipitipellis (S), and stipe context (SC). No significantly different taxa in the pileipellis (P) and lamellae (L); therefore they are not presented in the Lefse circle. White circles refer to taxa without significant differences among the five *Tricholoma matsutake* compartments.

An analysis of the main bacterial taxa explaining the differences of bacterial community across the *T. matsutake* compartments was performed using LEfSe analysis (Figure 5). No obvious biomarkers were statistically predicted in the pileipellis and lamellae of *T. matsutake*. The cladogram demonstrated that a total of 20 taxa showed significantly different abundances among the other compartments associated with *T. matsutake* fruiting bodies. There were 9, 8, and 3 bacterial taxa identified in stipitipellis (S), stipe context (SC), and pileum context (PC), respectively (Figure 5). The outer layer of *T. matsutake* (stipitipellis) was enriched with Micrococcales. In contrast, the inner layer was differentially enriched by other biomarkers: the stipe context was enriched with Bacillales, Deinococcus, Corynebacteriales, and Moraxellaceae, while taxa enriched in the pileum context (PC) were Caulobacter, Sphingomonas, and Cupriavidus (Figure 5).

### 3.4. Putative Function Change

**Figure 6:**
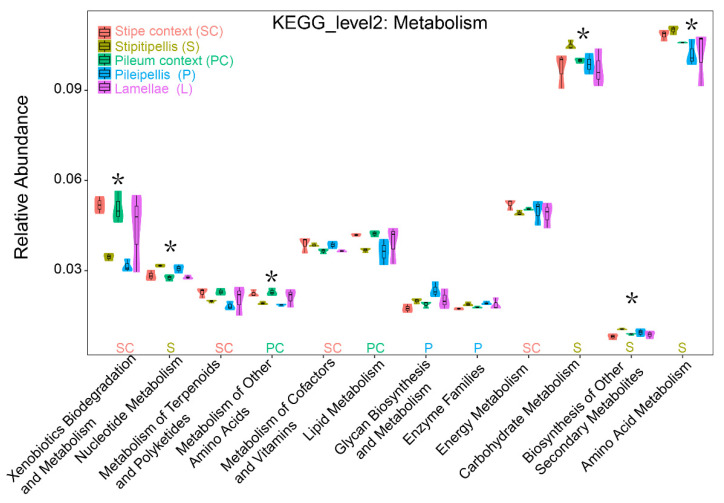
Change of microbial metabolism function profiles analyzed by PICRUSt. Abbreviations indicate the compartments of the *Tricholoma matsutake*: stipe context (SC), stipitipellis (S), pileum context (PC), pileipellis (P), lamellae (L). Asterisks indicate significant differences in the predicted metabolic function of the OUT-based microbial community analysis at 0.05 level by ANOVA, followed by Tukey HSD.

Putative function change showed that within the fruiting bodies of *T. matsutake*, processes involving amino acid and carbohydrate metabolism were the most abundant, followed by energy, xenobiotics biodegradation, and lipids (Figure 6). Predictive function analysis showed significant differences across the compartments of *T. matsutake*. Specifically, in the stipitipellis, where the Micrococcales were enriched, three potential metabolisms (amino acid, carbohydrate, and nucleotide), and biosynthesis of secondary metabolites were all highest (as predicted by PICRUSt; Figure 6) compared to other *T. matsutake* compartments; and in the pileipellis, the potential activities of enzyme families and glycan biosynthesis were the highest among the *T. matsutake* compartments. In contrast to the potential metabolisms in the *T. matsutake* outer layers (stipitipellis and pileipellis), the inner layer was highly enriched by other metabolic processes. For instance, in the stipe context (SC) where the Bacillales, Deinococcus, Corynebacteriales, and Moraxellaceae were biomarkers, the metabolisms of energy, cofactors, and vitamins were highest; and in the pileum context (PC) that was enriched with Caulobacter, Sphingomonas, and Cupriavidus, the metabolisms of lipids and amino acids were highest among the *T. matsutake* compartments (Figure 6). 

The KEGG ortholog homologous function predictions in *T. matsutake* fruiting bodies indicate that the highest three functional types (as shown by the relative gene-based function prediction) were an RNA polymerase sigma-70 factor (K03088), a 3-oxoacyl-[acyl-carrier protein] reductase (K00059), and a LacI family transcriptional regulator (K02529) (Table S1). In the *T. matsutake* inner layers, increased functional types shift to an aspartyl-tRNA(Asn)/glutamyl-tRNA (Gln) amidotransferase subunit A (K02433) and a cold shock protein (beta-ribbon, CspA family) (K03704), while in the outer layer of *T. matsutake*, multiple sugar transport system permease proteins (K02025 and K02026) were a strongly increased functional type (Table S4).

In the fungal ITS sequence analyses, after filtering out the *T. matsutake* sequences (559 OTUs, accounting for 63% of the total fungal OTUs), 319 fungal OTUs remained. Among these 319 non-host fungal OTUs, 219 fungal OTUs (68%) were assigned to specific trophic modes. Out of these functionally assigned OTUs, 111 OTUs with the “possible” confidence ranking were removed, and the remaining 40 and 68 OTUs that belonged to the acceptable confidence rankings assigned to the “highly probable” and “probable” types were used for downstream functional analysis.

**Figure 7:**
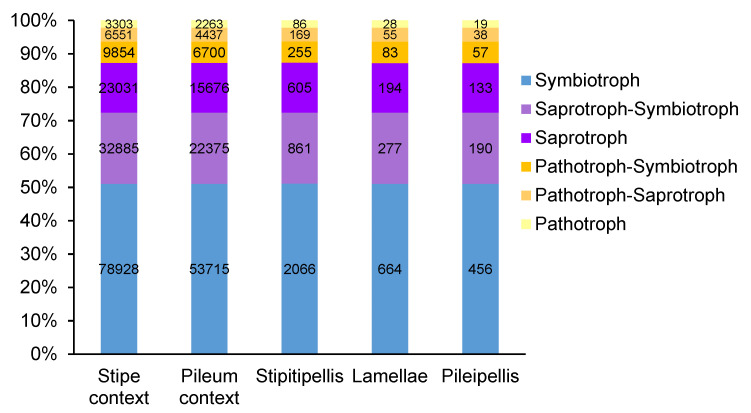
Relative abundance of fungal trophic modes among the different compartments of *Tricholoma matsutake* fruiting bodies. The within-column numbers indicate the absolute abundance of the representative OTUs belonging to each trophic mode.

Although the (within column) numbers of fungal OTUs were different, the relative abundance of fungal trophic modes was similar (Figure 7). The fungal trophic modes were dominated (~50%) by “Symbiotroph” type, followed by “Saprotroph” and “Saprotroph–Symbiotroph (~35%). The “pathotroph” type represented a relatively low abundance of less than 5%. Detailed information (name, guild, growth morphology, and trait) of fungal species is shown in Table S5.

## 4. Discussion

### 4.1. Differences in Microbial Attributes within T. matsutake Fruiting Body Structures

In support of our first hypothesis 1 (H1), both bacteria and fungi selectively inhabited different compartments of the *T. matsutake* fruiting body. Generally, more microbial species tended to inhabit the inner layer of *T. matsutake* as compared to the outer layer. This trend of higher microbial diversity at the inner layer was also found for another EMF, *Thelephora ganbajun* (also belonging to phylum Basidiomycota) [3]. 

Additionally, a more complex microbial partitioning was observed for the studied EMF within the micro-niches of the inner layers, and these niches include parts such as the stipe, pileum, and the lamellae. We expect this phenomenon is associated with microbial functioning. For instance, the pileum might be responsible for the development of adjoining compartments (lamellae and pileipellis), and may exhibit higher nutrient availability due to the associated growth-promoting bacteria [28]. 

In a recent investigation by Liu et al. (2021), while studying microbial functional gene compartmentalization in the EMF *T. ganbajun,* the thick interior context of the fungal fruiting body was found to have a greater abundance of C- and N-fixing genes, and sourcing energy and nutrition to the associated microbes [3]. Similarly, in the present study, the pileum context of *T. matsutake* may function as (i) an important reservoir of nutrients and (ii) a microbial hotspot niche as indicated by its highest microbial richness, in comparison to the other compartments of the fruiting body.

The context of the stipe also plays significant roles in providing structural support to the fruiting body skeleton as well as being a site of secondary metabolite production [29]. The quadrupled frequency of unique fungal OTUs within the stipe context compared to the remaining compartments of the fruiting body (Figure 2) is notable. These high-frequency fungal OTUs corresponded to those fungal endophytes, forming unique association within the *T. matsutake* fungal body, and may have greater biological relevance by assisting in secondary metabolite production [29,30]. Furthermore, secondary metabolites within the stipe context of *T. matsutake* could be enriched by—and enrich—N-fixing bacteria, as revealed by the much higher relative abundance of Moraxellaceae (belonging to the Pseudomonadales) in this micro-niche. The unique numbers of endofungal bacteria were much higher than those of the endofungal fungi. This higher prevalence of endofungal bacteria within the fruiting body of *T. matsutake* further elaborates their critical role in fruiting body differentiation/compartmentalization and is in line with the earlier study [25]. Microbiome community structure was significantly different among different *T. matsutake* compartments (H1). In particular, Bacteroidetes predominated in the outer layer of this EMF, while Proteobacteria were mainly localized in the inner layer. This highlights the role of Proteobacteria occupying micro-niches for nutrient acquisition and is in line with their copiotrophic life strategy [31,32]. Such associations may have been strengthened by the accumulation of carbohydrates, proteins, and lipids within the fungal fruiting body [33]. Although direct measurement of *T. matsutake* nutritional components (i.e., total sugar, crude protein, and fiber) was not included in our work, we observed significantly higher potential metabolisms involving vitamins, lipid, and amino acid synthesis in the inner layer of *T. matsutake* fruiting bodies in comparison to the compartments. Our results clearly show (Figure 2) that the different *T. matsutake* compartments showed unique fungal OTUs, e.g., the unique fungal OTUs found in the stipe context were around 4 times higher compared to those found in the other evaluated compartments (205 vs. 33 to 52). For bacteria, there were 499, 491, 450, 441, and 410 OTUs found exclusively in the pileum context, stipitipellis, stipe context, pileipellis, and lamellae, respectively. Meanwhile, for fungi there were 205, 52, 52, 47 and 33 exclusive OTUs for stipe context, pileum context, pileipellis, stipitipellis and lamellae, respectively. This shows a clear microbial compartmentalization in the studied maturation stage, which may be related to the biochemical, environmental and nutritional differential conditions in the evaluated microniches, i.e., changes in pH, CO_2_ concentration, volatile organic compounds, key odorants or nutritional composition [34,35]. However, we recognize that the microbial distribution is a dynamic process affected by the changes of these conditions, and therefore it would be very interesting to study the bacterial and fungal changes in the different compartments, which occurs during the whole basidiomata maturation, in order to have deeper and wider insights of the whole microbial dynamics, as it has previously been done for nutrient composition and volatile compounds [36]. Additionally, despite the fact that some light has been shed on the ascomata colonization process in truffles by microorganisms [37,38], the knowledge of the successional changes of the microbiome during the basidiomata formation since mycelial patches until senescence has so far remained unexplored, despite the fact that it constitutes a research area of great relevance.

### 4.2. Predictive Functional Profiling within T. matsutake Compartments

In agreement with our second hypothesis (H2), microbial potential metabolic functions varied among the *T. matsutake* compartments. Micro-niche partitioning disparity of the *T. matsutake* individual compartments (outer vs. inner layer), which assembles unique microbiomes, may explain this difference. Similar spatial niche-induced microbial recruitment and assembling trends were reported previously among fungus-associated components [39,40] and within fungus fruiting body parts [3,41]. Such strong and clear differences led us to explore the putative functions performed by the spatially distinct microbiomes in the EMF fungal fruiting body. In the present study, descriptions and inferred metabolic processes are focused on the potential prevalence of genes in the dominant microbes within each of the tissues. However, from a fungal ecology point of view, the *T. matsutake* mushrooms should be more considered as the host, with the endophytic and epiphytic microbes as either commensals and/or parasites that rely on the *T. matsutake* mushrooms to provide nutrients. Direct evidence for this is lacking, but preliminary potential functional profiles (using PICRUSt) support the idea of *T. matsutake* as a nutritional repository for endofungal microorganisms. Amino acid metabolism was the strongest identified function within the fruiting bodies of *T. matsutake.* As amino acids are the building blocks of proteins, polypeptides, and other nitrogenous biomolecules, their exchange between *T. matsutake* and its associated microbes could be of great importance. 

At the bacterial family classification level, we found that the Pseudomonadaceae and Sphingobacteriaceae showed higher relative abundances in the outer layer of the fruiting body. This could indicate their ecological roles in secreting antibiotics and substance-degrading enzymes such as hydrolases, chitinase, lysozyme, and xylanase [42,43]. There could be a dual effect from those endofungal bacteria because on the one hand, they can secrete antibiotics to defend against opportunist-microbes, and on the other hand those microbes secreting chitinases and glucanases are potentially useful for the decay of fruiting bodies after maturation. Although gene cluster coding for these antibacterial and antifungal compounds is not yet available on JGI (The Fungal Genomics Resource) or NCBI, preliminary results suggest that raw extracts from *T. matsutake* fruiting bodies (using 90% ethanol) possess moderate anti-bacterial properties against *Escherichia coli* (43%, suppression ratio), *Staphylococcus aureus* (38%), and *Salmonella enterica* (34%) [44]. 

Relative abundance of endofungal trophic modes within the *Tricholoma matsutake* fruiting bodies showed that the main endofungal fungi trophic mode was the symbiotroph (~50%, relative abundance) [45,46], with 31 fungal OTUs assigned to the *Thelephora, Tuber* and *Tomentella* genera. As these are also common EMF with strong infectivity, they could perform similar functions such as promoting phosphorus absorption and host growth as they do for host trees. Certain bacteria (e.g., *Stenotrophomonas* and *Ewingella*) were also differently presented in *T. matsutake* fruiting bodies, known as saprotrophic decomposers by secreting chitinases [13,19]. Compared to endofungal bacteria, the endofungal fungi exhibited more complex patterns, with 38 fungal OTUs assigned to 26 genera (occupying ~35% of overall trophic modes; Table S5) that belong to soil saprotroph [47], wood saprotroph [48], and dung saprotroph modes [49]. In the late stage of macrofungal morphogenesis, underground soil-derived opportunistic microbes can colonize fruiting bodies as pathogens [11]. For endofungal fungal communities, the pathotrophic fungi were only a small proportion (~10%) within the total fungal trophic modes. Among them, there were 16 detected fungal OTUs belonging to plant pathogens such as *Neofusicoccum*, *Mycosphaerella*, *Ascochyta*, *Podosphaera*, *Amphobotrys*, *Phaeoacremonium*, *Trichothecium*, *Cylindrocarpon*, *Gibberella*, *Slopeiomyces*, *Libertella*, *Monographella*, *Waitea*, *Anthracoidea*, and *Rhizophydium* [50]. In addition, some endofungal bacteria are also known as pathogens in mushroom breeding and cultivation areas, such as *Pseudomonas* species (i.e., *P. tolaasii* and *P. putida*) [51,52]. In the present study, *P. putida* was found in all *T. matsutake* compartments, with preference in the stipe context more than other parts (Table S6). Endofungal microorganisms showed complex associations (as either helpers, pathogens, or decomposers) with *T. matsutake* fruiting bodies, both in their different inhabiting patterns, diversity, and diverse roles.

To our knowledge, this is the first study of microbiome partitioning in fruiting bodies in an Agaricales fungus. The *T. matsutake* work is a seminal investigation whose strength is to show the diversity and specificity of microbiomes in different compartments. However, we have to recognize that the functional relevance and contribution of the microorganisms associated with *T. matsutake* fruiting bodies require deeper analysis with more powerful tools in the future.

## 5. Conclusions

As an important component of the terrestrial environmental, ectomycorrhizal fungi play vital roles in biogeochemical nutrient cycling in forest ecosystems. Based on our study of a selected model EMF, *Tricholoma matsutake*, we demonstrated that EMF do not merely function as fruiting bodies for spore dispersal, but also localize different microbial communities within their structures to generate microbial hotspots of varying importance and complexity. We have shown for the first time that in an Agaricales fruiting body, the fungus-related microbial environment is more complex than previously expected, in the context of not only the localized microbial taxa, but also based on their potential metabolic functions as well. Each compartment of the EMF fruiting body can be treated as a unique site of diverse secondary metabolite production, encompassing microbial hotspots with greater metabolic potential. Unlike the conventional macroscopic classification of environmental fungi, we explored an advanced approach of segregating the fungal fruiting body into its component parts, based on the associated microbes residing in each compartment. Such compartmentalization and associated metabolic functions therein can be explored more in light of the state-of-the-art techniques such as multi-omic (genomics, transcriptomics, proteomics, metabolomics etc.) approaches in future studies. 

## Figures and Tables

**Table 1:** Bacterial and fungal diversity indices of different compartments of *Tricholoma matsutake*.

	Bacteria				Fungi			
	Chao1	ACE	Simpson	Shannon	Chao1	ACE	Simpson	Shannon
SC	915 (18) c	943 (5) b	0.93 (0.03) a	6.25 (0.57) a	392 (85) a	389 (95) a	0.54 (0.16) a	2.66 (0.13) a
S	862 (24) c	863 (25) c	0.96 (0.02) a	6.75 (0.10) a	322 (29) a	325 (35) a	0.36 (0.17) b	1.92 (0.56) b
PC	1205 (63) a	1241 (67) a	0.94 (0.03) a	6.84 (0.42) a	343 (14) a	345 (14) a	0.38 (0.20) b	1.95 (0.60) b
P	914 (36) c	891 (76) c	0.95 (0.02) a	6.57 (0.64) a	325 (15) a	324 (18) a	0.42 (0.31) b	2.05 (0.26) b
L	960 (95) b	991 (85) b	0.93 (0.02) a	6.24 (0.83) a	345 (46) a	349 (47) a	0.24 (0.01) c	1.47 (0.01) c

Values shown are the means of three replicates with standard deviation (SD) in brackets. Values of same column followed by different lower-case letters are significantly different at *p* < 0.05 (ANOVA, Tukey HSD). Abbreviations: L = lamellae; P = pileipellis; PC = pileum context; S = stipitipellis; SC = stipe context.

## Data Availability

Not applicable.

## Supplementary Materials

The following are available online at https://www.mdpi.com/article/10.3390/jof7080586/s1. Table S1. The numbers of raw and clean reads for bacteria and fungi in the different compartments of *Trichioloma matsutake* fruiting bodies. Table S2. Bacterial OTUs matrix among the different compartments of *Trichioloma matsutake* fruiting bodies (L = lamellae; P = pileipellis; PC = pileum context; S = stipitipellis; SC = stipe context). Table S3. Fungal OTUs matrix among the different compartments of *Trichioloma matsutake* fruiting bodies (L = lamellae; P = pileipellis; PC = pileum context; S = stipitipellis; SC = stipe context). Table S4. Detailed assignments of KEGG ortholog homologous function predictions based on gene abundance. The four highlighted functional types are highest expressed functions among compartments of the *Tricholoma matsutake*—stipe context (SC), stipitipellis (S), pileum context (PC), pileipellis (P), lamellae (L). Table S5. Fungal OTUs and their trophic modes among the different compartments of *Trichioloma matsutake* fruiting bodies. Table S6. Potential bacterial species as pathogens or decomposers, among the different compartments of *Trichioloma matsutake* fruiting bodies (L = lamellae; P = pileipellis; PC = pileum context; S = stipitipellis; SC = stipe context). Figure S1. Relative abundance of the phylum (top 6 abundant) of bacteria found in different compartments associated with *Tricholoma matsutake* fruiting bodies. Figure S2. Relative abundance of the abundant fungal phylum found in different compartments associated with *Tricholoma matsutake* fruiting bodies. Abbreviations: L = lamellae; P = pileipellis; PC = pileum context; S = stipitipellis; SC = stipe context. Figure S3. Relative abundance of the abundant fungal phylum (A) and family (B) found in different compartments associated with *Tricholoma matsutake* fruiting bodies. Abbreviations: L = lamellae; P = pileipellis; PC = pileum context; S = stipitipellis; SC = stipe context.
